# Supplementary material for: The conserved RNA helicase YTHDC2 regulates the transition from proliferation to differentiation in the germline
Source: eLife. 2017 Oct 31;6:e26116. doi: 10.7554/eLife.26116 (PMC5703642; doi:10.7554/eLife.26116)
Supplement: Transparent reporting form [file elife-26116-transrepform.docx]

***­­­***

***eLife’s* transparent reporting form**

We encourage authors to provide detailed information *within their submission* to facilitate the interpretation and replication of experiments. If you have any questions, please contact us: [editorial@elifesciences.org](mailto:editorial@elifesciences.org).

**Sample-size estimation**

- You should state whether an appropriate sample size was computed when the study was being designed
- You should state the statistical method of sample size computation and any required assumptions
- If no explicit power analysis was used, you should describe how you decided what sample (replicate) size (number) to use

Please outline where this information can be found within the submission (e.g., page numbers or figure legends), or explain why this information doesn’t apply to your submission:

A description for how sample sizes were determined is provided in the “Statistics” section in the Materials and Methods. The sample sizes used for each experiment are provided in the Figure Legends.

**Replicates**

- You should report how often each experiment was performed
- You should include a definition of biological versus technical replication
- The data obtained should be provided and sufficient information should be provided to indicate the number of independent biological and/or technical replicates
- If you encountered any outliers, you should describe how these were handled
- Criteria for exclusion/inclusion of data should be clearly stated
- High-throughput sequence data should be uploaded before submission, with a private link for reviewers provided (these are available from both GEO and ArrayExpress)

Please outline where this information can be found within the submission (e.g., page numbers or figure legends), or explain why this information doesn’t apply to your submission:

RNA-Seq, m^6^A-IP and fRIP-Seq experiments were performed in biological replicates, i.e., samples were collected from different animals (described in the “Statistics” section of the Materials and Methods). Detailed methods of the RNA-Seq, m^6^A-IP and fRIP-Seq experiments are described in the Materials and Methods.

All Next generation sequencing is available at GEO, and programs used for data analysis are openly available. No data was excluded.

The private link for access to GEO data GSE93567: [https://www.ncbi.nlm.nih.gov/geo/query/acc.cgi?token=ufuryeoobvedrwx&acc=GSE93567](https://www.ncbi.nlm.nih.gov/geo/query/acc.cgi?token=ufuryeoobvedrwx&acc=GSE93567)

The link to the GEO data is also provided in the Materials and Methods section.

All phenotypic characterization experiments (both histology and immunofluorescence staining) were performed in multiple animals (n≥3) from independent crosses. Information regarding replicates is provided in the “Statistics” section of the Materials and Methods.

**Statistical reporting**

- Statistical analysis methods should be described and justified
- Raw data should be presented in figures whenever informative to do so (typically when N per group is less than 10)
- For each experiment, you should identify the statistical tests used, exact values of N, definitions of center, methods of multiple test correction, and dispersion and precision measures (e.g., mean, median, SD, SEM, confidence intervals; and, for the major substantive results, a measure of effect size (e.g., Pearson's r, Cohen's d)
- Report exact p-values wherever possible alongside the summary statistics and 95% confidence intervals. These should be reported for all key questions and not only when the p-value is less than 0.05.

The statistical tests used and the p-values are provided in the Results section as well as in the Figure Legends. The Figure Legends also provide the exact sample size (n) for each experiment.

Statistical tests are described in the “Statistics” section in the Materials and Methods. All information relevant to the tests used is reported.

Please outline where this information can be found within the submission (e.g., page numbers or figure legends), or explain why this information doesn’t apply to your submission:

(For large datasets, or papers with a very large number of statistical tests, you may upload a single table file with tests, Ns, etc., with reference to page numbers in the manuscript.)

**Additional data files (“source data”)**

- We encourage you to upload relevant additional data files, such as numerical data that are represented as a graph in a figure, or as a summary table
- Where provided, these should be in the most useful format, and they can be uploaded as “Source data” files linked to a main figure or table
- Include model definition files including the full list of parameters used
- Include code used for data analysis (e.g., R, MatLab)
- Avoid stating that data files are “available upon request”

Please indicate the figures or tables for which source data files have been provided:

Raw data and count data for RNA-Seq analysis are available from GEO. The link is provided in the manuscript in the “Data Availability” section of the Materials and Methods.

Source data files have been provided for Figure 2, Figure 3, Figure 4, Figure 5, Figure 6, Figure 7 and Figure 8.
